# Long-term efficacy and safety of left atrial appendage closure vs. oral anticoagulation in atrial fibrillation: a meta-analysis of randomized controlled trials

**DOI:** 10.1093/europace/euag159

**Published:** 2026-07-23

**Authors:** Paschalis Karakasis, Stylianos Tzeis, Athanasios Samaras, Konstantinos Vlachos, Konstantinos Pamporis, Panagiotis Theofilis, Apostolos Tzikas, Antonios P Antoniadis, Pierre Jaïs, Nikolaos Fragakis

**Affiliations:** Second Department of Cardiology, Hippokration General Hospital, Aristotle University of Thessaloniki, Thessaloniki, Greece; Department of Cardiology, Mitera Hospital, 6, Erythrou Stavrou Str. 151 23, Marousi, Athens, Greece; Second Department of Cardiology, Hippokration General Hospital, Aristotle University of Thessaloniki, Thessaloniki, Greece; Electrophysiology and Heart Modeling Institute, IHU Liryc, Fondation Bordeaux Université and Université de Bordeaux, INSERM U1045, Pessac, France; Cardiac Arrhythmia Department, INSERM U1045, CHU de Bordeaux, Pessac, France; Electrophysiology Department, Hygeia Hospital, Athens, Greece; First Department of Cardiology, School of Medicine, National and Kapodistrian University of Athens, Hippokration General Hospital, Athens, Greece; First Department of Cardiology, School of Medicine, National and Kapodistrian University of Athens, Hippokration General Hospital, Athens, Greece; Second Department of Cardiology, Hippokration General Hospital, Aristotle University of Thessaloniki, Thessaloniki, Greece; Second Department of Cardiology, Hippokration General Hospital, Aristotle University of Thessaloniki, Thessaloniki, Greece; Electrophysiology and Heart Modeling Institute, IHU Liryc, Fondation Bordeaux Université and Université de Bordeaux, INSERM U1045, Pessac, France; Cardiac Arrhythmia Department, INSERM U1045, CHU de Bordeaux, Pessac, France; Second Department of Cardiology, Hippokration General Hospital, Aristotle University of Thessaloniki, Thessaloniki, Greece

**Keywords:** Atrial fibrillation, Left atrial appendage closure, Oral anticoagulation, Stroke prevention, Thromboembolism, Ischemic stroke, Bleeding

## Abstract

**Aims:**

Left atrial appendage closure (LAAC) has emerged as an alternative to oral anticoagulation (OAC) for stroke prevention in atrial fibrillation (AF), but its long-term comparative efficacy remains uncertain. This systematic review and meta-analysis evaluated randomized controlled trials (RCTs) comparing LAAC with OAC in AF.

**Methods and results:**

MEDLINE, Embase, Scopus, and the Cochrane Database of Systematic Reviews were searched through 8 April 2026. Risk ratios (RR) with 95% confidence intervals (CI) were pooled using random-effects models. Certainty of evidence (CoE) was assessed using GRADE. Six RCTs involving 7004 participants were included (3681 assigned to LAAC and 3323 to OAC). Compared with OAC, LAAC resulted in a significantly higher risk of ischaemic stroke or systemic embolism (134 vs. 80 events; RR 1.41, 95% CI 1.07–1.86; moderate CoE). No significant differences were observed for any stroke or systemic embolism (RR 1.10, 95% CI 0.87–1.39; moderate CoE), all-cause mortality (RR 0.92, 95% CI 0.77–1.10; high CoE), cardiovascular mortality (RR 0.90, 95% CI 0.67–1.21; moderate CoE), non-cardiovascular mortality (RR 0.92, 95% CI 0.75–1.12; moderate CoE), major bleeding (RR 0.91, 95% CI 0.77–1.08; high CoE), or haemorrhagic stroke (RR 0.58, 95% CI 0.28–1.17; moderate CoE). LAAC reduced the risk of nonprocedural clinically relevant bleeding compared with OAC (RR 0.50, 95% CI 0.43–0.59; high CoE).

**Conclusion:**

In patients with AF, LAAC results in a higher risk of ischaemic stroke or systemic embolism than an OAC-based strategy. Compared with OAC, LAAC has no effect on major bleeding or mortality and does not reduce haemorrhagic stroke.

## Graphical Abstract

**Graphical Abstract:**
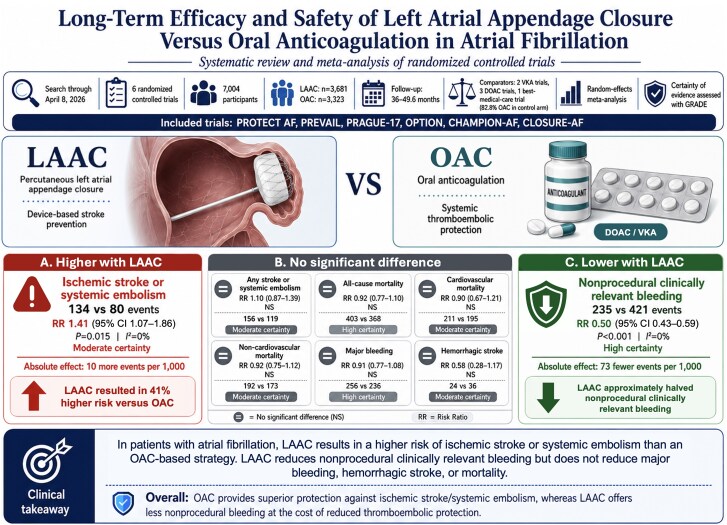

## Introduction

Atrial fibrillation (AF) is the most common sustained cardiac arrhythmia and a major cause of ischaemic stroke, systemic embolism, and mortality.^1,2^ Oral anticoagulation (OAC), preferably with a direct oral anticoagulant (DOAC) in eligible patients, remains the standard strategy for stroke prevention. Because most AF-related thrombi originate in the left atrial appendage, percutaneous left atrial appendage closure (LAAC) has emerged as a nonpharmacological alternative for patients with contraindications to long-term anticoagulation.^3–8^

Randomized evidence comparing LAAC with OAC has expanded considerably. The pivotal LAAC trials established the initial evidence base against warfarin,^9,10^ whereas more recent trials have evaluated LAAC against DOAC-based therapy in broader and more contemporary clinical settings.^11,12^ Most recently, CHAMPION-AF^13^ and CLOSURE-AF^14^ have further expanded the randomized evidence base, extending evaluation to patients at particularly high thromboembolic and bleeding risk. However, rather than resolving the question, these newer trials have made the overall signal more complex to interpret, with mixed findings across thromboembolic and bleeding outcomes.

Accordingly, we performed a systematic review and meta-analysis of all randomized controlled trials (RCTs), using the longest available follow-up, to define the long-term efficacy and safety of LAAC vs. OAC in patients with AF.

## Material and methods

This systematic review and meta-analysis were conducted in accordance with the Cochrane Handbook for Systematic Reviews^15^ and are reported in line with the PRISMA 2020 statement.^16^ The protocol was prospectively registered in the Open Science Framework (doi:10.17605/OSF.IO/5GK9Q) and adhered to without deviation. As the analysis was based exclusively on aggregate data from published studies, ethics approval and informed consent were not required.

### Search strategy

The search strategy was developed collaboratively by three investigators. MEDLINE (via PubMed), Embase, Scopus, and the Cochrane Database of Systematic Reviews were searched from inception to 8 April 2026, with no restrictions on language, publication year, publication status, or date of dissemination. Search syntax combined controlled vocabulary and free-text terms for left atrial appendage closure, atrial fibrillation, oral anticoagulation, and randomized trials. To increase retrieval sensitivity, supplementary searches were conducted in ClinicalTrials.gov, Epistemonikos, and Google Scholar, and backward and forward citation tracking was performed using the CitationChaser package in R.^17^ The full electronic search strategies are provided in Supplementary material online, *Tables S1*–S4.

### Eligibility criteria

#### Inclusion criteria

Eligible studies were RCTs enrolling adults with atrial fibrillation that directly compared percutaneous left atrial appendage closure with oral anticoagulation for stroke prevention. Trials were considered eligible if they reported at least one prespecified efficacy or safety outcome and provided sufficient data for quantitative synthesis.

#### Exclusion criteria

The following were excluded: non-comparative reports, including case reports, case series, and narrative reviews; non-original publications, including editorials, letters, commentaries, and expert opinion articles; and non-full-text or non-peer-reviewed sources, including conference abstracts, study protocols, clinical practice guidelines, and unpublished theses or dissertations. Observational studies and nonrandomized interventional studies were also excluded, as were cross-sectional, case-control, and crossover designs.

### Outcomes

The prespecified efficacy outcomes were any stroke or systemic embolism, ischaemic stroke or systemic embolism, all-cause mortality, cardiovascular mortality, and non-cardiovascular mortality. The prespecified safety outcomes were major bleeding, nonprocedural clinically relevant bleeding, and haemorrhagic stroke. All outcomes were extracted according to trial-level definitions (see Supplementary material online, *Table S5*).

### Study selection

Study selection was undertaken independently by three investigators in two stages. First, all retrieved records were screened by title and abstract. Records considered potentially eligible by at least one reviewer were advanced to full-text assessment. Second, full-text reports were evaluated independently against the predefined eligibility criteria to determine final inclusion. Disagreements were resolved by discussion and consensus, with adjudication by a senior author when required. Screening was performed using Abstrackr,^18^ and citation management and duplicate removal were undertaken in Mendeley.

### Data extraction

A standardized data extraction form was developed *a priori* and refined through pilot testing before formal extraction. Data extraction was performed independently by three investigators, with disagreements resolved by consensus and, when necessary, adjudication by a senior author.

Extracted data included study characteristics (first author, year of publication, country, enrolment period, study design, sample size, follow-up duration, treatment strategies, and postprocedural antithrombotic regimen), baseline participant characteristics (age, sex, stroke risk, bleeding risk, and relevant clinical comorbidities), and outcome data. Outcome-level data included endpoint definitions, effect estimates with corresponding 95% confidence intervals, and raw event counts where available. When more than one report was available for a given randomized trial, data extraction was based on the report providing the longest follow-up for the outcomes of interest. Where necessary, additional information was sought from the corresponding authors of the original reports.

### Quality assessment

The internal validity of the included RCTs was evaluated using the revised Cochrane Risk of Bias tool (RoB 2).^19^ Three reviewers independently assessed each trial across the five standard domains of bias, including the randomization process, deviations from intended interventions, missing outcome data, outcome measurement, and selective reporting. Overall study-level judgments were assigned according to the RoB 2 guidance. Trials were classified as low risk of bias only if all domains were rated as low risk. Any discrepancies were resolved by consensus, with arbitration by a third senior reviewer where required.

### Data analysis

All statistical analyses were performed in R (version 4.5.3). Trial-level dichotomous outcomes were pooled as risk ratios (RR) with 95% confidence intervals (CI) using random effects models. Separate meta-analyses were performed for each prespecified efficacy and safety outcome. Between-study variance was estimated under a frequentist framework using restricted maximum likelihood. Statistical significance was defined by a two-sided *P* value <0.05. For PROTECT AF and PREVAIL trials, the longest available outcomes were extracted from the 5-year patient-level pooled analysis reported by Reddy *et al*.^20^ Accordingly, these trials were entered into the primary meta-analysis as a single pooled PROTECT AF/PREVAIL comparison, consistent with the approach adopted in earlier meta-analysis.^21^

Statistical heterogeneity was quantified with the *I*^2^ statistic and assessed formally using Cochran's *Q* test. In keeping with conventional thresholds, *I*^2^ values of 0–30% were interpreted as low heterogeneity, 30–50% as moderate, 50–75% as substantial, and 75–100% as considerable heterogeneity.^22^

Prespecified subgroup analyses were conducted according to comparator type (VKA vs. DOAC), with subgroup differences assessed by interaction testing. Exploratory leave-one-out sensitivity analyses were also performed for the prespecified outcomes to assess whether any individual trial exerted a disproportionate influence on the pooled estimates. Small-study effects (including publication bias) were assessed visually using comparison-adjusted funnel plots and formally using Egger regression testing.

### Certainty of evidence assessment

The certainty of evidence for each outcome was evaluated using the Grading of Recommendations Assessment, Development and Evaluation (GRADE) approach, with disagreements resolved through discussion and adjudication by the senior reviewer. The assessment considered the principal GRADE domains, including risk of bias, inconsistency, imprecision, and indirectness. Certainty ratings and Summary of Findings table were generated using the GRADEpro Guideline Development Tool (GRADEpro GDT; McMaster University and Evidence Prime, 2024; https://www.gradepro.org/).

## Results

### Study selection and characteristics

**Figure 1:**
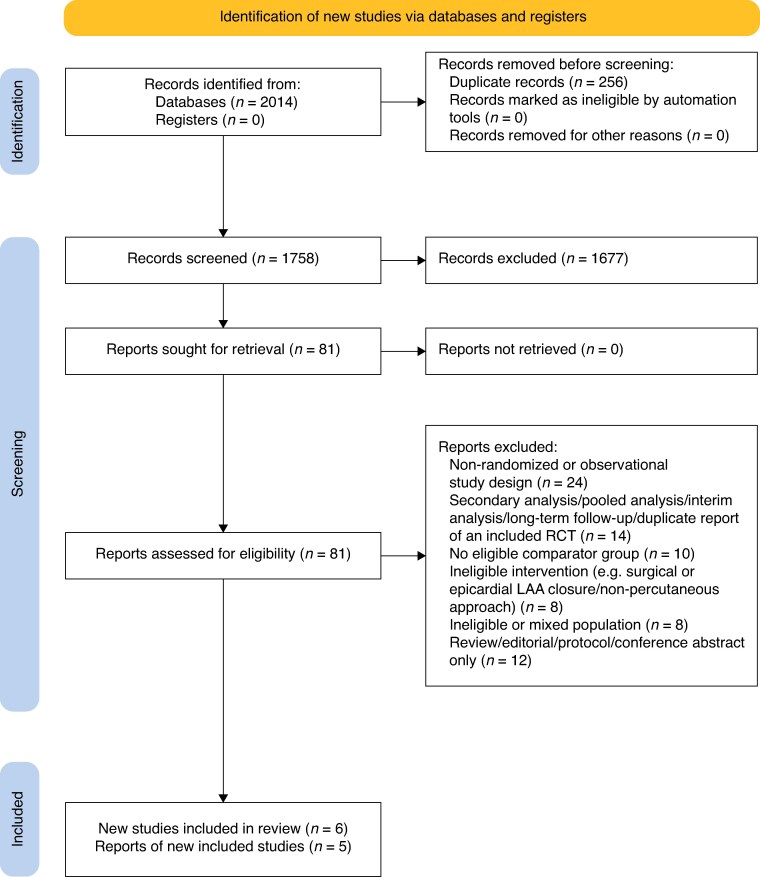
PRISMA 2020 flow diagram of study selection.

The study selection process is shown in the PRISMA 2020 flow diagram (*Figure 1*).

Overall, six randomized controlled trials were included,^11–14,20^ yielding five study comparisons for the pooled efficacy analyses and contributing data from 7004 participants, of whom 3681 were assigned to LAAC and 3323 to OAC. Two trials compared LAAC with VKA, three compared contemporary LAAC strategies with DOACs, and CLOSURE-AF compared LAAC with physician-directed best medical care. In CLOSURE-AF, of 442 patients assigned to medical therapy, 376 (85.1%) received a DOAC and 14 (3.2%) received a vitamin K antagonist, such that 390 patients (88.2%) were treated with OAC overall. As shown in Supplementary material online, *Figure S1*, trial participants were drawn predominantly from North America and Europe.

The main characteristics of the included trials are summarized in *Table 1*. Follow-up ranged from 36 to 49.6 months. Across the included trials, mean age ranged from 69.4 to 78.5 years, and women accounted for 25.4–38.7% of participants. Overall thromboembolic risk was moderate to high, with mean CHA₂DS₂-VASc scores ranging from 3.4 to 5.2; in the earlier LAAC vs. VKA trials, mean CHADS₂ scores ranged from 2.2 to 2.6. Bleeding risk varied across the more contemporary trials that reported it, with mean HAS-BLED scores ranging from 1.2 to 3.1; HAS-BLED was not reported in PROTECT AF or PREVAIL.

**Table 1:** Baseline characteristics of included randomized controlled trials comparing left atrial appendage closure vs. oral anticoagulation

Characteristic	PROTECT AF	PREVAIL	PRAGUE-17	OPTION	CHAMPION-AF	CLOSURE-AF
**Study characteristics**						
**First author-year**	Reddy VY—2014	Holmes DR Jr – 2014^10^	Osmancik P – 2022^11^	Wazni OM - 2024	Doshi SK – 2026^13^	Landmesser U - 2026^14^
**Enrolment**	2005–2008	2010–2012	2015–2019	2019–2020	NR	March 2018–May 2024
**Sample size (device vs. comparator)**	707 (463 vs. 244)	407 (269 vs. 138)	402 (201 vs. 201)	1600 (803 vs. 797)	3000 (1499 vs. 1501)	888 primary-analysis patients (446 vs. 442); 912 randomized
**Study design**	Non-inferiority	Non-inferiority	Non-inferiority	Non-inferiority	Non-inferiority	Non-inferiority
**Follow-up, months**	47.6	49.6	42	36	36	Median 36
**Treatment strategy**						
**Device group vs. comparator group**	WATCHMAN vs. VKA	WATCHMAN vs. VKA	Amulet, WATCHMAN, or WATCHMAN FLX vs. DOAC	WATCHMAN FLX vs. DOAC	WATCHMAN FLX vs. DOAC	Watchman / Watchman FLX / Amulet / LAmbre vs. physician-directed best medical care
**Antithrombotic regimen after LAAC implantation**	Aspirin for life; warfarin for 45 days; clopidogrel 45 days–6 months	Aspirin for life; warfarin for 45 days; clopidogrel 45 days–6 months	Aspirin for life; clopidogrel for first 3 months	OAC for first 3 months; aspirin for first 12 months	NOAC plus aspirin, NOAC monotherapy, or DAPT for first 3 months; then aspirin or P2Y12 monotherapy	DAPT ≥3 months, then aspirin to 6 months; shorter DAPT / aspirin monotherapy allowed in excessive bleeding risk
**Demographics and stroke\/bleeding risk**						
**Age, years**	71.7 ± 8.8 vs. 72.7 ± 9.2	74.0 ± 7.4 vs. 74.9 ± 7.2	73.4 ± 6.7 vs. 73.2 ± 7.2	69.7 ± 7.4 vs. 69.4 ± 7.9	71.6 ± 7.5 vs. 71.8 ± 7.5	78.5 ± 6.8 vs. 77.3 ± 7.3
**Male ratio (%)**	70.4 vs. 70.1	67.7 vs. 74.6	64.7 vs. 64.7	64.8 vs. 66.9	67.6 vs. 68.4	61.4 vs. 61.3
**White patient ratio (%)**	91.8 vs. 91.0	94.1 vs. 94.9	NR	83.8 vs. 86.1	85.1 vs. 85.0	93.0 vs. 94.1
**CHADS₂ score, mean (SD)**	2.2 ± 1.2 vs. 2.3 ± 1.2	2.6 ± 1.0 vs. 2.6 ± 1.0	NR	NR	NR	NR
**CHADS₂** **=** **1 (%)**	33.7 vs. 27.0	7.8 vs. 8.7	NR	NR	NR	NR
**CHADS₂** **=** **2 (%)**	34.1 vs. 36.1	50.9 vs. 44.9	NR	NR	NR	NR
**CHADS₂** **≥** **3 (%)**	32.2 vs. 32.2	41.3 vs. 46.4	NR	NR	NR	NR
**CHA₂DS₂-VASc score, mean (SD)**	3.4 ± 1.5 vs. 3.7 ± 1.6	3.8 ± 1.2 vs. 3.9 ± 1.2	4.7 ± 1.5 vs. 4.7 ± 1.5	3.5 ± 1.3 vs. 3.5 ± 1.3	3.5 ± 1.2 vs. 3.5 ± 1.3	5.2 ± 1.5 vs. 5.1 ± 1.6
**CHA₂DS₂-VASc ≤3 (%)**	NR	36.1 vs. 37.7	24.9 vs. 23.9	53.3 vs. 52.9	52.3 vs. 59.1	13.7 vs. 15.6
**CHA₂DS₂-VASc 4–5 (%)**	NR	53.9 vs. 52.2	48.3 vs. 48.3	38.6 vs. 38.5	37.7 vs. 33.9	44.4 vs. 44.1
**CHA₂DS₂-VASc ≥6 (%)**	NR	10.0 vs. 10.9	26.9 vs. 27.9	7.5 vs. 7.7	10.1 vs. 7.0	41.9 vs. 40.3
**Risk factors for stroke (%)**						
**Heart failure**	26.8 vs. 27.0	23.4 vs. 23.2	44.8 vs. 43.8	NR	NR	NR
**History of hypertension**	89.6 vs. 90.2	88.5 vs. 97.1	92.5 vs. 92.5	NR	NR	93.5 vs. 94.3
**Age ≥75 years**	41.0 vs. 47.1	52.0 vs. 56.5	39.3 vs. 42.3	NR	NR	74.2 vs. 67.2
**Diabetes**	24.4 vs. 29.5	33.8 vs. 29.7	44.8 vs. 36.3	NR	NR	39.2 vs. 42.1
**Previous ischaemic stroke or TIA (%)**	17.7 vs. 20.1	27.5 vs. 28.3	34.3 vs. 36.3	NR	7.9 vs. 7.6^a^	31.9 vs. 34.2^b^
**HAS-BLED score, mean (SD)**	NR	NR	3.0 ± 0.9 vs. 3.1 ± 0.9	1.2 ± 0.8 vs. 1.2 ± 0.8	1.3 ± 0.8 vs. 1.3 ± 0.8	3.1 ± 0.9 vs. 3.0 ± 0.9
**Atrial fibrillation profile**						
**Atrial fibrillation pattern: paroxysmal (%)**	43.2 vs. 40.6	48.7 vs. 51.4	33.3 vs. 26.4	40.6 vs. 37.1	69.3 vs. 68.5	NR
**Atrial fibrillation pattern: persistent (%)**	21.0 vs. 20.5	31.6 vs. 28.3	30.9 vs. 32.4	59.4 vs. 62.9	23.9 vs. 25.4	NR
**Atrial fibrillation pattern: permanent (%)**	34.6 vs. 38.1	15.6 vs. 15.9	35.8 vs. 41.3	NR	6.8 vs. 6.1	NR

**Abbreviations**: AF, atrial fibrillation; CHADS₂, congestive heart failure, hypertension, age ≥75 years, diabetes mellitus, prior stroke or transient ischaemic attack (2 points); CHA₂DS₂-VASc, congestive heart failure, hypertension, age ≥75 years (2 points), diabetes mellitus, prior stroke or transient ischaemic attack/systemic embolism (2 points), vascular disease, age 65–74 years, sex category (female); HAS-BLED, hypertension, abnormal renal/liver function, stroke, bleeding history or predisposition, labile international normalized ratio, elderly, drugs/alcohol; DAPT, dual antiplatelet therapy; DOAC, direct oral anticoagulant; OAC, oral anticoagulation; LAAC, left atrial appendage closure; SD, standard deviation; TIA, transient ischaemic attack; VKA, vitamin K antagonist; NR, not reported; P2Y12, P2Y12 receptor inhibitor; vs., versus.

^a^CHAMPION-AF reported previous ischaemic stroke rather than a combined ischaemic stroke/TIA baseline row.

^b^CLOSURE-AF reported stroke or transient ischaemic attack.

Risk-of-bias assessments for the included randomized trials are shown in Supplementary material online, *Figure S2*; all trials were judged to be of some concern overall.

### Efficacy outcomes

Five study comparisons (six RCTs) contributed to each efficacy outcome, representing 7004 participants overall (3681 assigned to LAAC and 3323 to OAC).

**Figure 2:**
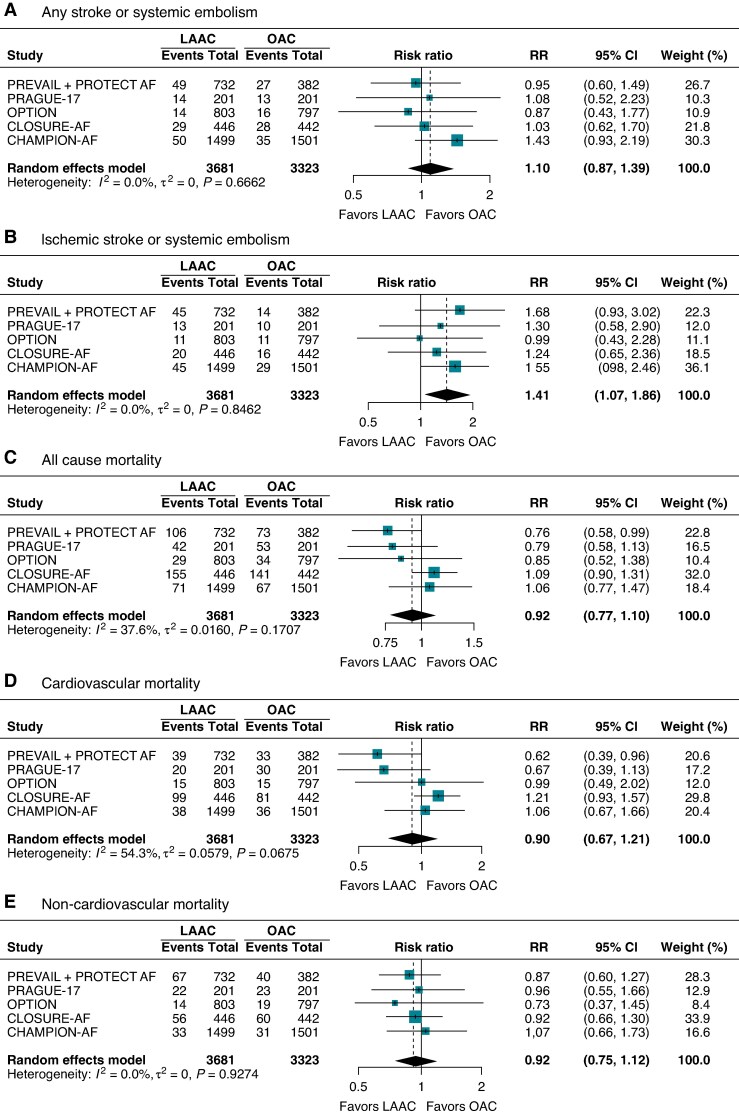
Long-term efficacy outcomes of left atrial appendage closure vs. oral anticoagulation in atrial fibrillation. Forest plots summarize trial-specific and pooled risk ratios for any stroke or systemic embolism, ischaemic stroke or systemic embolism, all-cause mortality, cardiovascular mortality, and non-cardiovascular mortality. Squares indicate individual trial estimates, with area proportional to weight, and horizontal lines indicate 95% confidence intervals; diamonds indicate pooled estimates from random effects models. Values less than 1.0 favour left atrial appendage closure. LAAC = left atrial appendage closure; OAC = oral anticoagulation; RR = risk ratio.

For any stroke or systemic embolism, 275 events were recorded overall, with 156 events in the LAAC group and 119 in the OAC group. There was no significant difference between treatment strategies (RR 1.10, 95% CI 0.87–1.39; *P* = 0.44; *I*^2^ = 0.0%; *Figure 2A*).

For ischaemic stroke or systemic embolism, 214 events were recorded overall, with 134 events in the LAAC group and 80 in the OAC group. LAAC was associated with a significantly higher risk of ischaemic stroke or systemic embolism than OAC (RR 1.41, 95% CI 1.07–1.86; *P* = 0.015; *I*^2^ = 0.0%; *Figure 2B*).

For all-cause mortality, 771 events were recorded overall, with 403 events in the LAAC group and 368 in the OAC group. There was no significant difference between treatment strategies (RR 0.92, 95% CI 0.77–1.10; *P* = 0.37; *I*^2^ = 37.6%; *Figure 2C*).

For cardiovascular mortality, 406 events were recorded overall, with 211 events in the LAAC group and 195 in the OAC group. There was no significant difference between treatment strategies (RR 0.90, 95% CI 0.67–1.21; *P* = 0.50; *I*^2^ = 54.3%; *Figure 2D*).

For non-cardiovascular mortality, 365 events were recorded overall, with 192 events in the LAAC group and 173 in the OAC group. There was no significant difference between treatment strategies (RR 0.92, 95% CI 0.75–1.12; *P* = 0.40; *I*^2^ = 0.0%; *Figure 2E*).

### Safety outcomes

**Figure 3:**
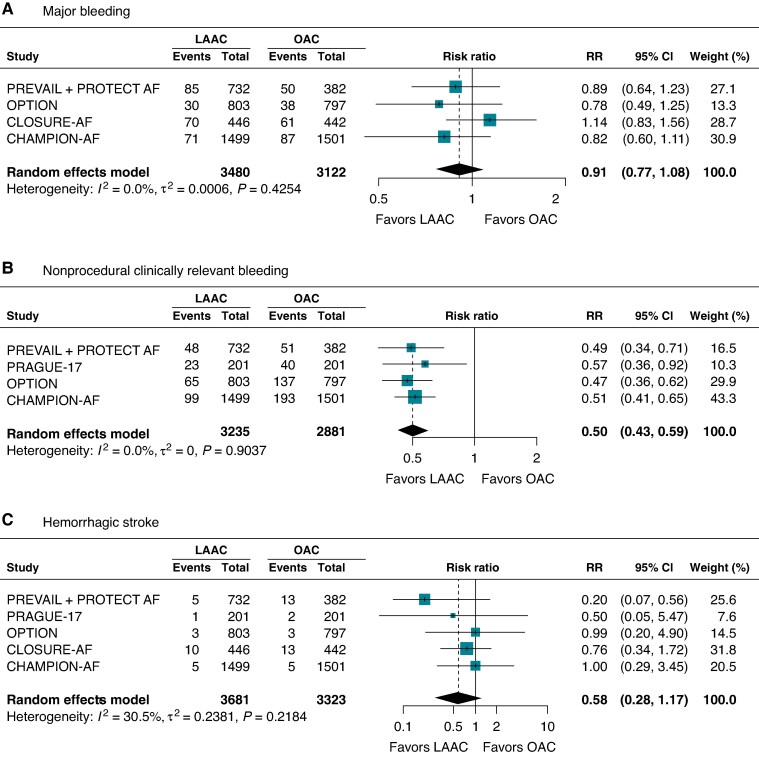
Safety outcomes of left atrial appendage closure vs. oral anticoagulation in atrial fibrillation. Forest plots show major bleeding, non-procedural clinically relevant bleeding, and haemorrhagic stroke. Squares represent trial-specific risk ratios, with area proportional to study weight, and horizontal lines indicate 95% confidence intervals; diamonds represent pooled estimates from random effects models. Values less than 1.0 favour left atrial appendage closure. LAAC = left atrial appendage closure; OAC = oral anticoagulation; RR = risk ratio.

For major bleeding, four study comparisons were included in the meta-analysis, representing 6602 participants overall, including 3480 assigned to LAAC and 3122 assigned to OAC. A total of 492 events were recorded, with 256 events in the LAAC group and 236 in the OAC group. There was no significant difference between treatment strategies (RR 0.91, 95% CI 0.77–1.08; *P* = 0.30; *I*^2^ = 0.0%; *Figure 3A*).

For nonprocedural clinically relevant bleeding, four study comparisons were included, representing 6116 participants overall, including 3235 assigned to LAAC and 2881 assigned to OAC. A total of 656 events were recorded, with 235 events in the LAAC group and 421 in the OAC group. LAAC was associated with a significantly lower risk of nonprocedural clinically relevant bleeding than OAC (RR 0.50, 95% CI 0.43–0.59; *P* < 0.001; *I*^2^ = 0.0%; *Figure 3B*).

For haemorrhagic stroke, five study comparisons were included, representing 7004 participants overall, including 3681 assigned to LAAC and 3323 assigned to OAC. A total of 60 events were recorded, with 24 events in the LAAC group and 36 in the OAC group. There was no significant difference between treatment strategies (RR 0.58, 95% CI 0.28–1.17; *P* = 0.13; *I*^2^ = 30.5%; *Figure 3C*).

### Subgroup analysis according to OAC type

Subgroup analyses according to comparator type showed no significant interaction for any efficacy outcome (all *P* for interaction >0.05; Supplementary material online, *Figure S3*).

For safety outcomes, no subgroup differences were observed for major bleeding or nonprocedural clinically relevant bleeding, with consistent estimates across VKA- and DOAC-based trials (see Supplementary material online, *Figure S4*). For haemorrhagic stroke, a significant interaction was observed (*P* for interaction = 0.02), with LAAC associated with a lower risk in VKA-based trials (RR 0.20, 95% CI 0.07–0.56) but not in DOAC-based trials (RR 0.82, 95% CI 0.45–1.51).

### Leave-one-out sensitivity analysis

Exploratory leave-one-out sensitivity analyses did not identify any single clearly influential or outlying study. The pooled estimates for any stroke or systemic embolism, all-cause mortality, cardiovascular mortality, non-cardiovascular mortality, major bleeding, and haemorrhagic stroke remained non-significant after sequential omission of individual trials. For ischaemic stroke or systemic embolism, the direction of effect consistently favoured OAC across all leave-one-out analyses, although statistical significance was attenuated when either the combined PROTECT AF/PREVAIL comparison or CHAMPION-AF was omitted, consistent with the limited number of RCTs and reduced statistical power after exclusion of large contributing studies. The reduction in non-procedural clinically relevant bleeding with LAAC remained robust in all leave-one-out analyses (see Supplementary material online, *Figure S5*).

### Small study effects and publication bias

Qualitative inspection of the contour-enhanced funnel plots did not suggest a consistent pattern of asymmetry, and Egger's regression tests were non-significant across the assessed outcomes, providing no evidence of small-study effects or publication bias (see Supplementary material online, *Figure S6*).

### Certainty of evidence

The certainty of evidence for the prespecified outcomes is presented in the Summary of Evidence table (*Table 2*). Certainty was rated as high for all-cause mortality, major bleeding, and non-procedural clinically relevant bleeding, and as moderate for any stroke or systemic embolism, ischaemic stroke or systemic embolism, cardiovascular mortality, non-cardiovascular mortality, and haemorrhagic stroke. Downgrading to moderate certainty was driven by imprecision, reflecting moderate event numbers and confidence intervals that did not exclude clinically important differences between treatment strategies.

**Table 2:** Summary of findings and certainty of evidence for left atrial appendage closure vs. oral anticoagulation in patients with atrial fibrillation

Outcomes	No of participants (studies) Follow-up	Certainty of the evidence (GRADE)	Relative effect (95% CI)	Anticipated absolute effects	
				Risk with OAC	Risk difference with LAAC*
Any stroke or systemic embolism	7004 (6 RCTs)	⨁⨁⨁◯ Moderate^a^	**RR 1.10** (0.87 to 1.39)	36 per 1.000	**4 more per 1.000** (from 5 fewer to 14 more)
Ischaemic stroke or systemic embolism	7004 (6 RCTs)	⨁⨁⨁◯ Moderate^a^	**RR 1.41** (1.07 to 1.86)	24 per 1.000	**10 more per 1.000** (from 2 more to 21 more)
All-cause mortality	7004 (6 RCTs)	⨁⨁⨁⨁ High	**RR 0.92** (0.77 to 1.10)	111 per 1.000	**9 fewer per 1.000** (from 25 fewer to 11 more)
Cardiovascular mortality	7004 (6 RCTs)	⨁⨁⨁◯ Moderate^b^	**RR 0.90** (0.67 to 1.21)	59 per 1.000	**6 fewer per 1.000** (from 19 fewer to 12 more)
Non-cardiovascular mortality	7004 (6 RCTs)	⨁⨁⨁◯ Moderate^b^	**RR 0.92** (0.75 to 1.12)	52 per 1.000	**4 fewer per 1.000** (from 13 fewer to 6 more)
Major bleeding	6602 (5 RCTs)	⨁⨁⨁⨁ High	**RR 0.91** (0.77 to 1.08)	84 per 1.000	**8 fewer per 1.000** (from 19 fewer to 7 more)
Non-procedural clinically relevant bleeding	6116 (5 RCTs)	⨁⨁⨁⨁ High	**RR 0.50** (0.43 to 0.59)	146 per 1.000	**73 fewer per 1.000** (from 83 fewer to 60 fewer)
Haemorrhagic stroke	7004 (6 RCTs)	⨁⨁⨁◯ Moderate^c^	**RR 0.58** (0.28 to 1.17)	11 per 1.000	**5 fewer per 1.000** (from 8 fewer to 2 more)

^*^The risk in the intervention group (and its 95% confidence interval) is based on the assumed risk in the comparison group and the relative effect of the intervention (and its 95% CI).

CI: confidence interval; RR: risk ratio.

^a^Downgraded one level for imprecision because the pooled estimate was based on a moderate number of events and the 95% CI overlapped RR 1.25, so a clinically important difference between LAAC and OAC could not be excluded.

^b^Downgraded one level for imprecision because the pooled estimate was based on a moderate number of events and the 95% CI overlapped RR 0.75, so a clinically important difference between LAAC and OAC could not be excluded.

^c^Downgraded one level for imprecision because haemorrhagic stroke was a rare outcome with few events and a wide 95% CI crossing no effect, including substantial benefit but not ruling out any important difference.

## Discussion

### Main findings and comparison with prior evidence

This meta-analysis assembles the totality of randomized evidence to date—six RCTs and 7004 patients—comparing percutaneous LAAC with OAC in AF. Four findings emerged: (i) compared with OAC, LAAC resulted in 41% higher relative risk of ischaemic stroke or systemic embolism (RR 1.41, 95% CI 1.07–1.86); (ii) there are no significant differences for any stroke or systemic embolism, nor for all-cause, cardiovascular, or non-cardiovascular mortality; (iii) LAAC does not reduce major bleeding or haemorrhagic stroke overall; and (iv) LAAC approximately halves the rate of non-procedural clinically relevant bleeding (RR 0.50, 95% CI 0.43–0.59).

When expressed as anticipated absolute effects, LAAC corresponded to approximately 10 additional ischaemic stroke or systemic embolism events per 1000 patients and 73 fewer non-procedural clinically relevant bleeding events per 1000 patients over 3–4 years of follow-up. This corresponds to an approximate number needed to harm of 100 for ischaemic thromboembolic events and an approximate number needed to treat of 14 for nonprocedural clinically relevant bleeding.

These findings update the most recent RCT-only meta-analysis by Kaisaier *et al*.^21^ which included four RCTs and 3116 patients before the publication of CHAMPION-AF and CLOSURE-AF. In that analysis, LAAC was associated with lower all-cause mortality, cardiovascular or unexplained mortality, haemorrhagic stroke, and non-procedural clinically relevant bleeding, without a significant difference in ischaemic stroke or systemic embolism. By incorporating the two newest RCTs, the present meta-analysis more than doubles the randomized sample size and includes the most recent DOAC-era and physician-directed medical-therapy comparisons. In this updated evidence base, the previously reported reductions in mortality and haemorrhagic stroke were no longer observed, whereas the reduction in nonprocedural clinically relevant bleeding remained consistent. In contrast, ischaemic stroke or systemic embolism was significantly more frequent with LAAC than with OAC.

### Mechanistic basis of residual ischaemic thromboembolic risk after LAAC

The higher risk of ischaemic stroke or systemic embolism with LAAC is mechanistically plausible. OAC provides systemic, source-agnostic thromboembolic protection, whereas LAAC is an anatomically restricted intervention that mitigates embolism arising from the LAA. Although the observation that most non-valvular AF thrombi originate in the appendage has provided the foundation for LAAC,^23^ contemporary concepts of AF-related thrombogenesis increasingly emphasize a broader atrial substrate. The recent EHRA/HRS/APHRS/LAHRS consensus on atrial cardiomyopathy^24^ frames thromboembolism as a manifestation of diffuse atrial disease, characterized by fibrosis, mechanical dysfunction, endothelial abnormalities, and a prothrombotic milieu.^25,26^ Accordingly, the temporal dissociation between AF episodes and embolic events,^27,28^ the association between impaired LA function and cerebrovascular events independent of AF,^29^ and the frequent coexistence of LAA thrombus with broader atrial myopathy^30^ support a model in which the appendage is often dominant, but not exclusive, as a thromboembolic source. Within this framework, OAC may provide more comprehensive protection against AF-related thrombogenesis, whereas LAAC addresses only the principal anatomic site of clot formation.

Device-based therapy also carries residual liabilities that are absent from pharmacological anticoagulation, including peri-procedural stroke, device embolization, device-related thrombosis (DRT), and peridevice leaks (PDL).^31–35^ In the present analysis, these complications were uncommon and appeared less frequent in contemporary trials (see Supplementary material online, *Table S6*), consistent with improvements in device design, imaging, and procedural experience. Nevertheless, DRT was reported in trials with systematic follow-up imaging, and residual PDL remained detectable in a proportion of patients, although large PDL were rare. These findings suggest that the mechanical limitations of LAAC have diminished over time but have not been eliminated. This residual device-related risk, together with the inability of LAAC to protect against non-LAA thromboembolic mechanisms, may help explain why LAAC did not match OAC for ischaemic stroke or systemic embolism.

### Interpretation across heterogeneous trial settings

The pooled estimates should be interpreted in light of important trial-level heterogeneity. The included RCTs span almost two decades and differ in device generation, procedural era, imaging guidance, post-procedural antithrombotic therapy, comparator strategy, and enrolled population. Early studies compared first-generation LAAC with VKA therapy, whereas contemporary trials evaluated newer device platforms against DOAC-based or physician-directed medical therapy.^11–14,20^ The clinical settings also differed: OPTION enrolled patients after AF ablation, CHAMPION-AF studied anticoagulation-eligible patients treated with a single contemporary device platform, and CLOSURE-AF enrolled an older, higher-risk population with individualized device and antithrombotic strategies.

These differences do not weaken the randomized nature of the evidence, and statistical heterogeneity was low for most outcomes. However, they indicate that the pooled estimates represent an average comparative effect across related but non-identical clinical scenarios, rather than a single effect that can be applied uniformly to all LAAC candidates. This is particularly relevant when interpreting apparent temporal trends. Although procedural safety and device performance have improved over time, contemporary refinement has not clearly translated into superior thromboembolic efficacy. In CHAMPION-AF, ischaemic stroke or systemic embolism occurred more frequently with LAAC than with DOAC therapy despite use of a contemporary device and high rates of effective closure.^13^ Similarly, the haemorrhagic stroke and mortality advantages suggested in earlier warfarin-era evidence were not consistently reproduced in DOAC-era trials. Thus, comparator type, trial era, and patient phenotype remain central to interpreting the balance of benefit and harm for LAAC vs. OAC. The importance of patient profile was clearly outlined by a cohort study that enrolled 1020 patients with a mean age of 79 years.^36^ The study identified specific predictors of 2-year mortality on the basis of baseline profile, including age, heart failure, vascular disease, valvular disease, abnormal liver and renal functions, with mortality rising as the number of risk factors increased, reaching 46.1% in patients presenting with five or six risk factors.^36^

### Differential effects on major and clinically relevant bleeding

LAAC did not reduce major bleeding compared with OAC, a finding supported by high-certainty evidence. However, LAAC reduced nonprocedural clinically relevant bleeding by approximately 50%, with no observed heterogeneity, indicating a consistent reduction in less severe but clinically consequential bleeding events. This pattern likely reflects the different temporal profiles of treatment-related bleeding: early procedure-related bleeding and short-term post-implant antithrombotic therapy may offset reductions in major bleeding, whereas avoidance of long-term OAC reduces later non-procedural bleeding. Accordingly, major bleeding and nonprocedural clinically relevant bleeding should be interpreted as distinct outcomes, the former capturing severe bleeding events and the latter reflecting a broader component of long-term treatment burden that may influence OAC adherence, discontinuation, and patient preference.^6,37^

### Contemporary clinical positioning of LAAC

The cumulative evidence does not support positioning LAAC as an interchangeable substitute for OAC in unselected patients with AF. Rather, its most evidence-consistent role remains in carefully selected patients in whom long-term OAC is unsuitable because of absolute contraindications, recurrent bleeding, or comorbidities that preclude sustained anticoagulation, as reflected in current guideline recommendations.^3,38,39^ Importantly, the randomized trials included in this meta-analysis largely enrolled anticoagulation-eligible patients; therefore, the evidence base for the population most commonly considered for LAAC in practice remains partly indirect. In this context, ASAP-TOO^40^ is expected to provide important complementary evidence by evaluating LAAC in patients unsuitable for OAC, a population that is highly relevant to contemporary clinical practice but distinct from the LAAC-vs.-OAC comparison addressed in the present meta-analysis.

A more selective role may also exist in patients with thromboembolism despite adequate OAC, in whom appendage closure could address a residual embolic source not fully mitigated by systemic anticoagulation. Observational data support this hypothesis,^40^ but randomized confirmation is needed. More broadly, LAAOS III showed that appendage occlusion can reduce stroke when added to background anticoagulation, supporting a potential complementary rather than purely substitutive relationship between appendage exclusion and systemic anticoagulation.^41–43^ Whether this synergistic concept extends to percutaneous LAAC is being evaluated in dedicated trials of LAAO plus OAC vs. OAC alone, including LAAOS-IV^44^ and ELAPSE trial.^45^ In parallel, recent evidence from a network meta-analysis of post-LAAO antithrombotic strategies suggests that DOAC-based regimens may offer a favourable safety–efficacy profile after device implantation, further emphasizing that outcomes after LAAC are shaped not only by appendage exclusion itself but also by the accompanying pharmacological strategy.^46^ Thus, the future role of LAAC may depend on phenotype-specific selection and, in selected high-risk patients, complementary use with anticoagulation rather than broad substitution for OAC.

### Strengths and limitations

This study has several important strengths. To the best of our knowledge, it represents the most comprehensive meta-analysis of randomized trials comparing percutaneous LAAC with OAC for long-term stroke prevention in AF, incorporating the full spectrum of contemporary evidence. Methodologically, the meta-analysis was conducted according to a prospectively registered protocol, a comprehensive systematic search, standardized risk-of-bias assessment, and strict adherence to Cochrane methodological recommendations. In addition, certainty of evidence was assessed using GRADE, and the conclusions were formulated in accordance with Cochrane's suggested statements for conclusions informed by the GRADE certainty-of-evidence assessment, thereby enhancing the transparency, consistency, and interpretability of the findings.

Several limitations should be acknowledged. First, this was a trial-level meta-analysis and therefore could not examine patient-level effect modifiers, including age, sex, thromboembolic risk, bleeding history, renal function, frailty, or prior stroke, nor could it support subgroup analyses across these clinically relevant characteristics. This limitation is particularly relevant for specific high-risk settings, such as end-stage renal disease or dialysis dependency, in which comparative evidence for LAAC vs. alternative stroke-prevention strategies remains limited to observational data.^47^ Second, there was substantial heterogeneity in the definition of composite endpoints across the included trials, which precluded quantitative synthesis of these outcomes. Third, procedural complications and device-related issues unique to LAAC, such as device embolization, pericardial effusion, and PDL, represent important treatment-related harms and should be considered in the overall safety assessment. However, these front-loaded procedural and device-related risks are not directly comparable to the continuously distributed treatment-related risks of OAC, which may complicate interpretation of the comparative safety profile.

An additional consideration relates to CLOSURE-AF, in which 88.2% of patients in the control arm received OAC, whereas the remainder did not. This may have diluted the comparator arm in the pooled efficacy analysis. For ischaemic stroke or systemic embolism, however, such dilution would be expected to attenuate the between-group difference, as a fully OAC-treated control arm would likely have experienced fewer events and therefore yielded a larger treatment effect in favour of OAC.

## Conclusions

In patients with AF, LAAC results in a higher risk of ischaemic stroke or systemic embolism than an OAC-based strategy. Compared with OAC, LAAC has no effect on major bleeding or mortality and does not reduce haemorrhagic stroke.

## Supplementary Material

euag159_Supplementary_Data

## Data Availability

The data underlying this article will be shared on reasonable request to the corresponding author. The items outlined in the PRISMA 2020 checklist and their corresponding locations within the manuscript are presented in Supplementary material online, *Table S7*.

## Protocol

The study protocol was prospectively registered in OSF (https://doi.org/10.17605/OSF.IO/5GK9Q).
