# Supplementary material for: Long-term efficacy and safety of left atrial appendage closure vs. oral anticoagulation in atrial fibrillation: a meta-analysis of randomized controlled trials
Source: Europace. 2026 Jul 23;28(7):euag159. doi: 10.1093/europace/euag159 (PMC13391209; doi:10.1093/europace/euag159)
Supplement: euag159_Supplementary_Data [file euag159_supplementary_data.docx]

**Supplementary material**

**Long-Term Efficacy and Safety of Left Atrial Appendage Closure Versus Oral Anticoagulation in Atrial Fibrillation: A Meta-Analysis of Randomized Controlled Trials**

Contents

[Supplemental Table 1. Search strategy for MEDLINE (Pubmed) 2](#_Toc230086066)

[Supplemental Table 2. Search strategy for Embase 2](#_Toc230086067)

[Supplemental Table 3. Search strategy for Cochrane library 3](#_Toc230086068)

[Supplemental Table 4. Search strategy for Scopus 4](#_Toc230086069)

[Supplemental Table 5. Original endpoint definitions across randomized trials 5](#_Toc230086070)

[Supplemental Figure 1. Geographic distribution of trial participants across included randomized trials 7](#_Toc230086071)

[Supplemental Figure 2. Risk-of-bias assessment of included randomized controlled trials using the RoB 2 tool. 8](#_Toc230086072)

[Supplemental Figure 3. Subgroup analysis of efficacy outcomes according to oral anticoagulant type. 10](#_Toc230086073)

[Supplemental Figure 4. Subgroup analysis of safety outcomes according to oral anticoagulant type. 11](#_Toc230086074)

[Supplemental Figure 5. Leave-one-out sensitivity analyses for efficacy and safety outcomes. 12](#_Toc230086075)

[Supplemental Figure 6. Contour-enhanced funnel plots for assessment of small-study effects. 13](#_Toc230086076)

[Supplemental Table 6. LAAC Procedural- or Device-Related Complications in Included Trials. 14](#_Toc230086077)

[Supplemental Table 7. PRISMA 2020 Checklist 15](#_Toc230086078)

# **Supplemental Table 1.** Search strategy for MEDLINE (Pubmed)

| ("Atrial Fibrillation"[MeSH Terms] OR "Atrial Fibrillation"[Title/Abstract] OR "AF"[Title/Abstract]) AND ("left atrial appendage"[Title/Abstract] OR "left atrial appendage closure"[Title/Abstract] OR "left atrial appendage occlusion"[Title/Abstract] OR "left atrial appendage closure device"[Title/Abstract] OR "left atrial appendage occlud*"[Title/Abstract] OR "LAA"[Title/Abstract] OR "LAAC"[Title/Abstract] OR "LAAO"[Title/Abstract] OR "WATCHMAN"[Title/Abstract] OR "Watchman FLX"[Title/Abstract] OR "Amulet"[Title/Abstract] OR "Amplatzer Cardiac Plug"[Title/Abstract] OR "LAmbre"[Title/Abstract]) AND ("Anticoagulants"[MeSH Terms] OR "Warfarin"[MeSH Terms] OR "Rivaroxaban"[MeSH Terms] OR "Dabigatran"[MeSH Terms] OR "anticoag*"[Title/Abstract] OR "oral anticoag*"[Title/Abstract] OR "OAC"[Title/Abstract] OR "OACs"[Title/Abstract] OR "DOAC"[Title/Abstract] OR "DOACs"[Title/Abstract] OR "NOAC"[Title/Abstract] OR "NOACs"[Title/Abstract] OR "Warfarin"[Title/Abstract] OR "vitamin k antagonist*"[Title/Abstract] OR "VKA"[Title/Abstract] OR "VKAs"[Title/Abstract] OR "apixaban"[Title/Abstract] OR "Rivaroxaban"[Title/Abstract] OR "Dabigatran"[Title/Abstract] OR "edoxaban"[Title/Abstract] OR "best medical care"[Title/Abstract] OR "medical therap*"[Title/Abstract]) AND ("randomized controlled trial"[Publication Type] OR "controlled clinical trial"[Publication Type] OR "random*"[Title/Abstract] OR "randomly"[Title/Abstract] OR "trial"[Title/Abstract] OR "placebo"[Title/Abstract]) |
| --- |

# **Supplemental Table 2.** Search strategy for Embase

| 1. exp left atrial appendage/  2. ((left atrial appendage or left atrial auricle) adj3 (closure or occlusion or occlud* or clos* or device*)).ti,ab,kw.  3. LAAC.ti,ab,kw.  4. LAAO.ti,ab,kw.  5. WATCHMAN.ti,ab,kw.  6. "Watchman FLX".ti,ab,kw.  7. Amulet.ti,ab,kw.  8. "Amplatzer Cardiac Plug".ti,ab,kw.  9. LAmbre.ti,ab,kw.  10. 1 or 2 or 3 or 4 or 5 or 6 or 7 or 8 or 9  11. exp atrial fibrillation/  12. atrial fibrillation.ti,ab,kw.  13. 11 or 12  14. exp anticoagulant agent/  15. exp warfarin/  16. exp apixaban/  17. exp rivaroxaban/  18. exp dabigatran etexilate/  19. exp edoxaban/  20. anticoag*.ti,ab,kw.  21. oral anticoag*.ti,ab,kw.  22. warfarin.ti,ab,kw.  23. "vitamin K antagonist*".ti,ab,kw.  24. VKA.ti,ab,kw.  25. VKAs.ti,ab,kw.  26. apixaban.ti,ab,kw.  27. rivaroxaban.ti,ab,kw.  28. dabigatran.ti,ab,kw.  29. edoxaban.ti,ab,kw.  30. DOAC.ti,ab,kw.  31. DOACs.ti,ab,kw.  32. NOAC.ti,ab,kw.  33. NOACs.ti,ab,kw.  34. "direct oral anticoagulant*".ti,ab,kw.  35. "non-vitamin K antagonist oral anticoagulant*".ti,ab,kw.  36. "novel oral anticoagulant*".ti,ab,kw.  37. 14 or 15 or 16 or 17 or 18 or 19 or 20 or 21 or 22 or 23 or 24 or 25 or 26 or 27 or 28 or 29 or 30 or 31 or 32 or 33 or 34 or 35 or 36  38. randomized controlled trial/  39. clinical trial/  40. random*.ti,ab,kw.  41. randomization.ti,ab,kw.  42. randomly.ti,ab,kw.  43. placebo*.ti,ab,kw.  44. trial.ti.  45. 38 or 39 or 40 or 41 or 42 or 43 or 44  46. 10 and 13 and 37 and 45 |
| --- |

# **Supplemental Table 3.** Search strategy for Cochrane library

| ID Search  #1 "left atrial appendage"  #2 "left atrial appendage closure"  #3 "left atrial appendage occlusion"  #4 "left atrial appendage closure device"  #5 "left atrial appendage occlud*"  #6 LAAC  #7 LAAO  #8 WATCHMAN  #9 "Watchman FLX"  #10 Amulet  #11 "Amplatzer Cardiac Plug"  #12 LAmbre  #13 atrial fibrillation  #14 AF  #15 MeSH descriptor: [Atrial Fibrillation] explode all trees  #16 anticoag*  #17 "oral anticoag*"  #18 OAC  #19 OACs  #20 warfarin  #21 "vitamin K antagonist*"  #22 VKA  #23 VKAs  #24 apixaban  #25 rivaroxaban  #26 dabigatran  #27 edoxaban  #28 DOAC  #29 DOACs  #30 NOAC  #31 NOACs  #32 "direct oral anticoagulant*"  #33 "non-vitamin K antagonist oral anticoagulant*"  #34 "novel oral anticoagulant*"  #35 "best medical care"  #36 "medical therap*"  #37 MeSH descriptor: [Anticoagulants] explode all trees  #38 MeSH descriptor: [Warfarin] explode all trees  #39 MeSH descriptor: [Apixaban] explode all trees  #40 MeSH descriptor: [Rivaroxaban] explode all trees  #41 MeSH descriptor: [Dabigatran] explode all trees  #42 MeSH descriptor: [Edoxaban] explode all trees  #43 random*  #44 trial  #45 placebo  #46 #1 OR #2 OR #3 OR #4 OR #5 OR #6 OR #7 OR #8 OR #9 OR #10 OR #11 OR #12  #47 #13 OR #14 OR #15  #48 #16 OR #17 OR #18 OR #19 OR #20 OR #21 OR #22 OR #23 OR #24 OR #25 OR #26 OR #27 OR #28 OR #29 OR #30 OR #31 OR #32 OR #33 OR #34 OR #35 OR #36 OR #37 OR #38 OR #39 OR #40 OR #41 OR #42  #49 #43 OR #44 OR #45  #50 #46 AND #47 AND #48 AND #49 |
| --- |

# **Supplemental Table 4.** Search strategy for Scopus

| TITLE-ABS-KEY(("left atrial appendage" OR "left atrial appendage closure" OR "left atrial appendage occlusion" OR "left atrial appendage closure device" OR "left atrial appendage occlud*" OR LAAC OR LAAO OR WATCHMAN OR "Watchman FLX" OR Amulet OR "Amplatzer Cardiac Plug" OR LAmbre) AND ("atrial fibrillation" OR AF) AND (anticoag* OR "oral anticoag*" OR OAC OR OACs OR warfarin OR "vitamin K antagonist*" OR VKA OR VKAs OR apixaban OR rivaroxaban OR dabigatran OR edoxaban OR DOAC OR DOACs OR NOAC OR NOACs OR "direct oral anticoagulant*" OR "non-vitamin K antagonist oral anticoagulant*" OR "novel oral anticoagulant*" OR "best medical care" OR "medical therap*") AND (random* OR trial OR placebo)) |
| --- |

# **Supplemental Table 5.** Original endpoint definitions across randomized trials

| Trial | Any stroke / SE | Ischemic stroke / SE | Major bleeding | Nonprocedural clinically relevant bleeding | Hemorrhagic stroke |
| --- | --- | --- | --- | --- | --- |
| CLOSURE-AF | Stroke = ischemic or hemorrhagic stroke; SE = abrupt vascular insufficiency of an extremity or organ with clinical/radiological evidence of arterial occlusion and no other likely mechanism. | Ischemic stroke = acute focal cerebral, spinal, or retinal dysfunction caused by CNS infarction; hemorrhagic transformation classified as ischemic stroke. | Major bleeding = BARC type 3 or higher | Not reported | Hemorrhagic stroke = acute focal/global cerebral or spinal dysfunction caused by intraparenchymal, intraventricular, or subarachnoid hemorrhage. |
| CHAMPION-AF | Stroke = ischemic or hemorrhagic stroke; SE = acute arterial occlusion of an extremity or non-CNS organ with clinical/imaging/surgical/autopsy confirmation. | Ischemic stroke = focal cerebral, spinal, or retinal infarction; ischemic stroke/SE was an additional primary efficacy endpoint planned at 5 years. | ISTH major bleeding; secondary safety endpoint included both procedure-related and nonprocedural ISTH major bleeding. | Modified ISTH criteria for clinically relevant nonmajor bleeding. | Hemorrhagic stroke included intracerebral or subarachnoid hemorrhage; hemorrhagic transformation of ischemic stroke classified as ischemic stroke. |
| OPTION | Stroke or SE formed part of the primary efficacy endpoint with all-cause death; SE = acute vascular insufficiency/occlusion of an extremity or non-CNS organ with clinical, imaging, surgical, or autopsy evidence and no other likely mechanism. | Ischemic stroke = neurological dysfunction caused by focal cerebral, spinal, or retinal infarction; ischemic stroke/SE was reported among additional clinical endpoints. | ISTH major bleeding; secondary endpoint included major bleeding through 36 months, including procedure-related bleeding. | Bleeding that required medical intervention, led to hospitalization or increased level of care, or prompted a face-to-face evaluation | Hemorrhagic stroke included intracerebral or subarachnoid hemorrhage; hemorrhagic transformation of ischemic stroke classified as ischemic stroke. |
| PRAGUE-17 | Stroke/TIA or SE formed part of the primary composite endpoint; stroke = sudden nontraumatic focal neurological deficit in a major cerebral artery territory, classified as ischemic, hemorrhagic, or unspecified; SE = acute vascular occlusion of an extremity or organ documented by imaging, surgery, or autopsy. | Ischemic stroke was included within all-stroke/TIA; ischemic stroke/SE was extractable from reported stroke subtype and SE events. | Clinically significant major bleeding defined according to ISTH criteria: overt bleeding with hemoglobin decrease ≥20 g/L, transfusion ≥2 units, critical-site bleeding, or fatal bleeding. | Clinically relevant bleeding = ISTH major bleeding + clinically relevant nonmajor bleeding; nonmajor bleeding = bleeding requiring hospitalization or invasive procedure. | Hemorrhagic stroke was included within all-stroke/TIA and categorized as a stroke subtype. |
| PREVAIL | First co-primary efficacy endpoint: composite of ischemic or hemorrhagic stroke, SE, and cardiovascular/unexplained death. | Second co-primary efficacy endpoint: ischemic stroke or SE occurring >7 days after randomization. | ISTH major bleeding criteria | Hemorrhage not meeting ISTH major bleeding criteria but requiring medical intervention by a healthcare professional or leading to hospitalization or increased level of care. | Included within the first co-primary efficacy endpoint as part of all-stroke. |
| PROTECT AF | Primary effectiveness endpoint: all stroke, including ischemic and hemorrhagic stroke, SE, and cardiovascular/unexplained death. | Ischemic stroke was reported as a stroke subtype; SE was included in the primary effectiveness endpoint. TIA was a secondary endpoint, defined as an acute focal neurological event lasting ≥5 minutes and ≤24 hours with negative MR imaging. | ISTH major bleeding criteria | Hemorrhage not meeting ISTH major bleeding criteria but requiring medical intervention by a healthcare professional or leading to hospitalization or increased level of care. | Included within all-stroke and reported separately as hemorrhagic stroke. |


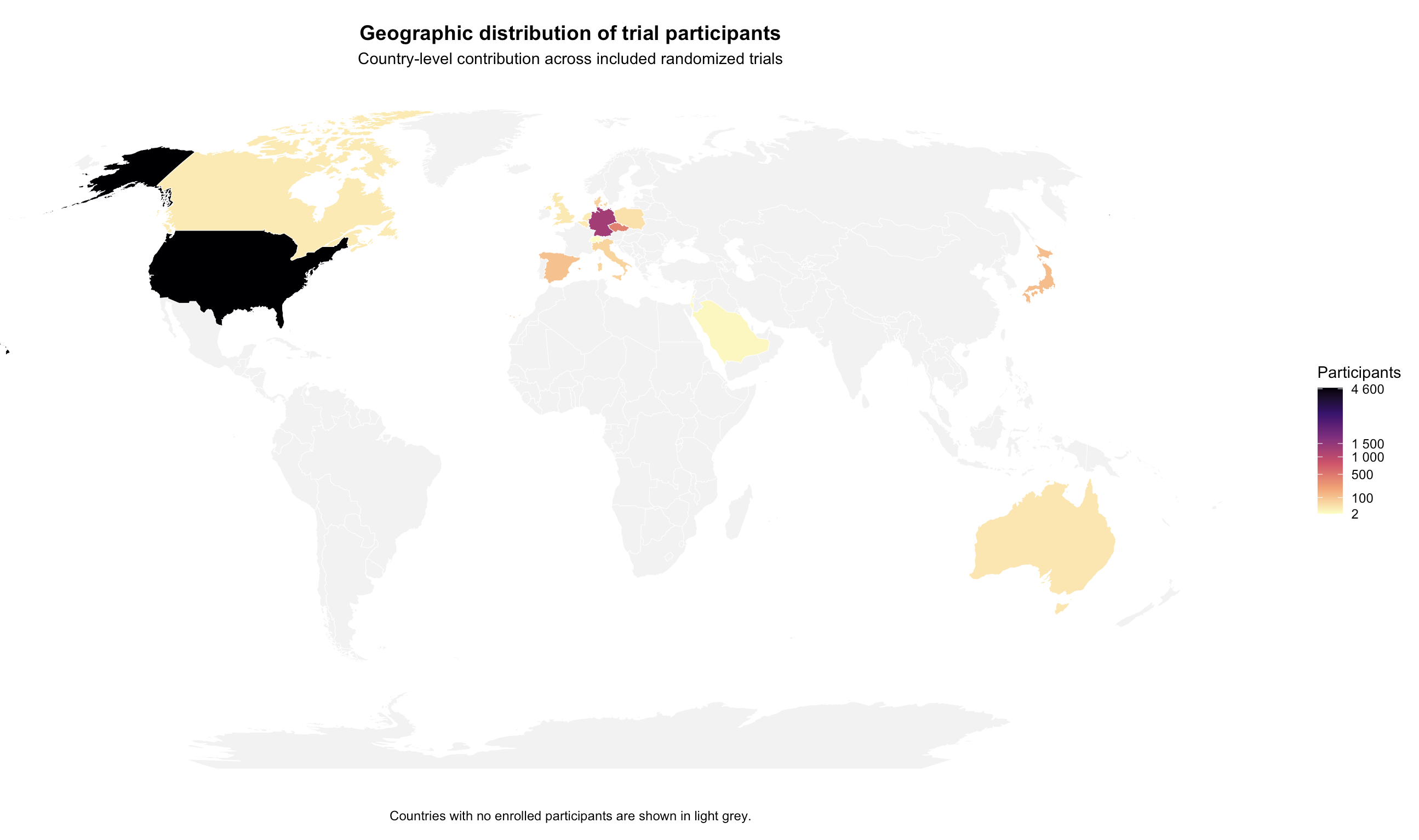


**Supplemental Figure 1**. Geographic distribution of trial participants across included randomized trials. World map showing the country-level contribution of randomized participants across the trials included in the analysis. Countries are shaded according to the cumulative number of participants contributed, with darker colors indicating greater enrollment. Countries with no enrolled participants are shown in light grey.


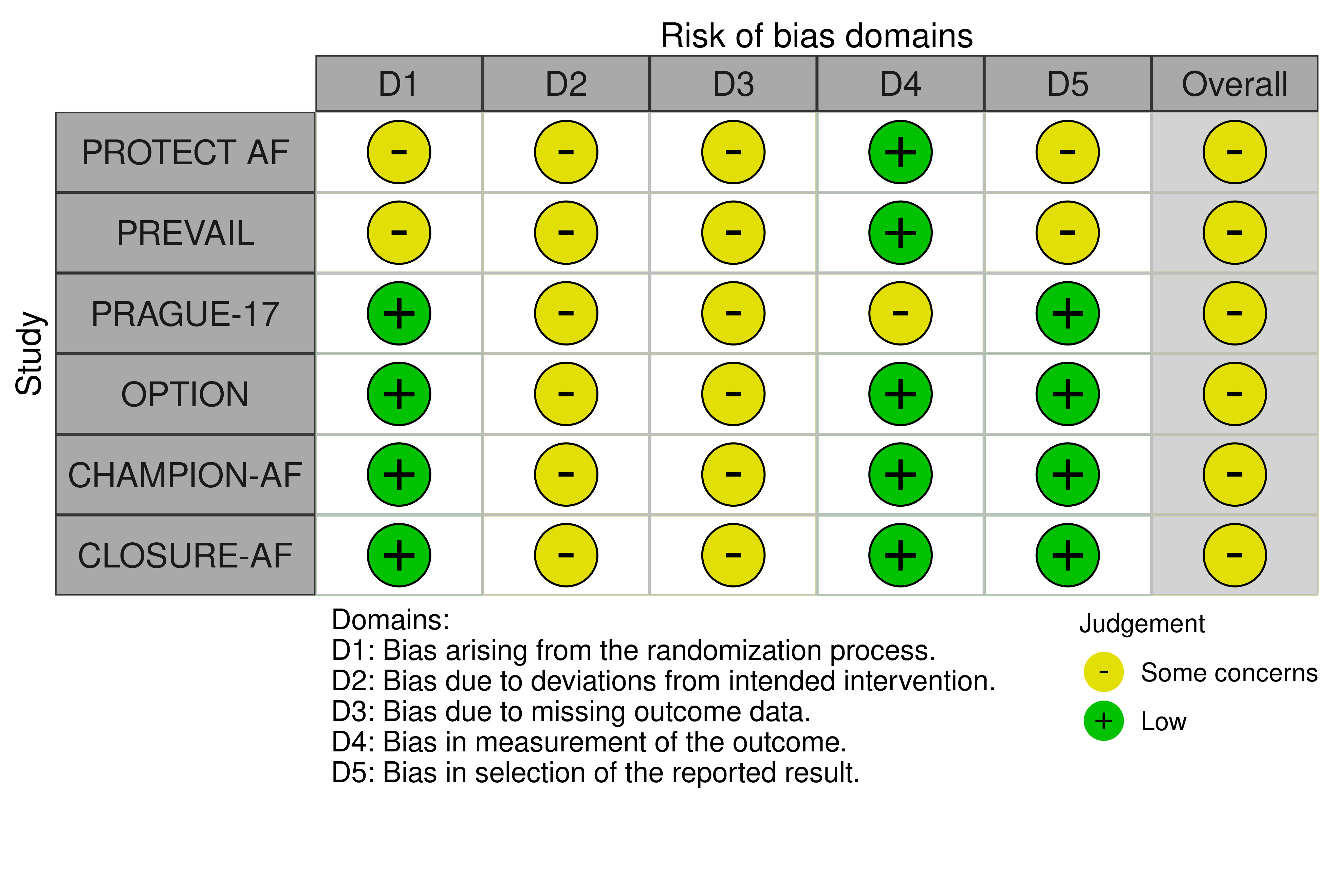


**Supplemental Figure 2**. Risk-of-bias assessment of included randomized controlled trials using the RoB 2 tool. Risk of bias was evaluated across five domains: D1, bias arising from the randomization process; D2, bias due to deviations from intended interventions; D3, bias due to missing outcome data; D4, bias in measurement of the outcome; and D5, bias in selection of the reported result. Green circles with a plus sign indicate low risk of bias, whereas yellow circles with a minus sign indicate some concerns.

PROTECT AF: D1: SC — computer randomization reported, but allocation-concealment details were limited; one site was censored for data-quality concerns. D2: SC — OL device-vs-warfarin strategy; 55 LAAC-assigned pts did not receive device, while post-LAAC Tx changed according to TEE findings; ITT and postprocedure/per-protocol analyses partially mitigated this. D3: SC — WD was differential and substantial, especially in warfarin arm, with censoring at last known status; informative attrition could not be fully excluded. D5: SC — Bayesian sequential design with repeated analyses, broad NI margin, and nominal long-term/secondary analyses without multiplicity adjustment; selective emphasis could not be fully excluded.

PREVAIL: D1: SC — computerized/centralized randomization was reported, but 2:1 allocation with site-stratified blocks and a small control arm led to some baseline imbalance; allocation-process details were not sufficient for unequivocal LR. D2: SC — OL device-vs-warfarin strategy; protocol-driven Tx changes in LAAC arm and aborted/nonattempted implants occurred, while warfarin management required active monitoring. D3: SC — 18-mo endpoint estimation relied on incomplete observed FU for some pts, with adaptive/Bayesian modelling using all available FU. D5: SC — multiple coprimary endpoints/Bayesian priors from PROTECT AF/CAP and no multiplicity adjustment; selective emphasis on endpoints meeting NI/safety criteria could not be fully excluded.

PRAGUE-17: D2: SC — OL LAAC-vs-DOAC strategy; individualized post-LAAC antithrombotic Tx, nonreceipt/failed LAAC, DOAC discontinuation, and CO occurred; mITT primary analysis in a NI design may bias toward null, although PP/on-Tx analyses were concordant. D3: SC — incomplete protocol-mandated follow-up imaging due to COVID-19, limiting systematic ascertainment of late device-related findings. D4: SC — outcome assessment was not clearly fully blinded; bleeding and procedure/device-related events may be partly influenced by OL Tx awareness and differential surveillance.

OPTION: D2: SC — OL device-vs-OAC strategy; 50 LAAC-assigned pts did not receive device and 82 OAC-assigned pts crossed to LAAC; OAC exposure also differed over FU, although ITT plus PP/on-Tx sensitivity analyses mitigated this. D3: SC — incomplete 36-mo FU with WD/LTFU in both arms, numerically greater in OAC; no imputation, with censoring at last FU, so informative censoring could not be fully excluded.

CHAMPION-AF: D2: SC — unblinded device-vs-NOAC strategy; post-LAAC antithrombotic Tx varied; nonreceipt of device and CO to LAAC occurred, with lower sustained Tx adherence in the LAAC arm, although ITT analysis mitigated this. D3: SC — incomplete 3-year endpoint status/attrition was present and greater in the NOAC arm; MI and sensitivity analyses were performed, but informative censoring could not be fully excluded.

CLOSURE-AF: D2: SC — OL pragmatic design; Tx exposure and post-LAAC antithrombotic regimen were physician-directed; CO/nonreceipt of allocated intervention occurred, although mitigated by ITT analysis and blinded CEC adjudication. D3: SC — WD/LTFU were present and partly imbalanced, with no planned imputation; informative censoring or event masking by non-CV death could not be fully excluded.

Abbreviations: SC, some concerns; LR, low risk; OL, open-label; Tx, treatment; pts, patients; LAAC, left atrial appendage closure; OAC, oral anticoagulation; NOAC, non-vitamin K antagonist oral anticoagulant; DOAC, direct oral anticoagulant; warfarin, vitamin K antagonist therapy; CO, crossover; ITT, intention-to-treat; mITT, modified intention-to-treat; PP, per-protocol; on-Tx, on-treatment; NI, noninferiority; FU, follow-up; WD, withdrawal; LTFU, lost to follow-up; MI, multiple imputation; TEE, transesophageal echocardiography; CEC, clinical events committee; CAP, Continued Access PROTECT AF Registry; CV, cardiovascular.


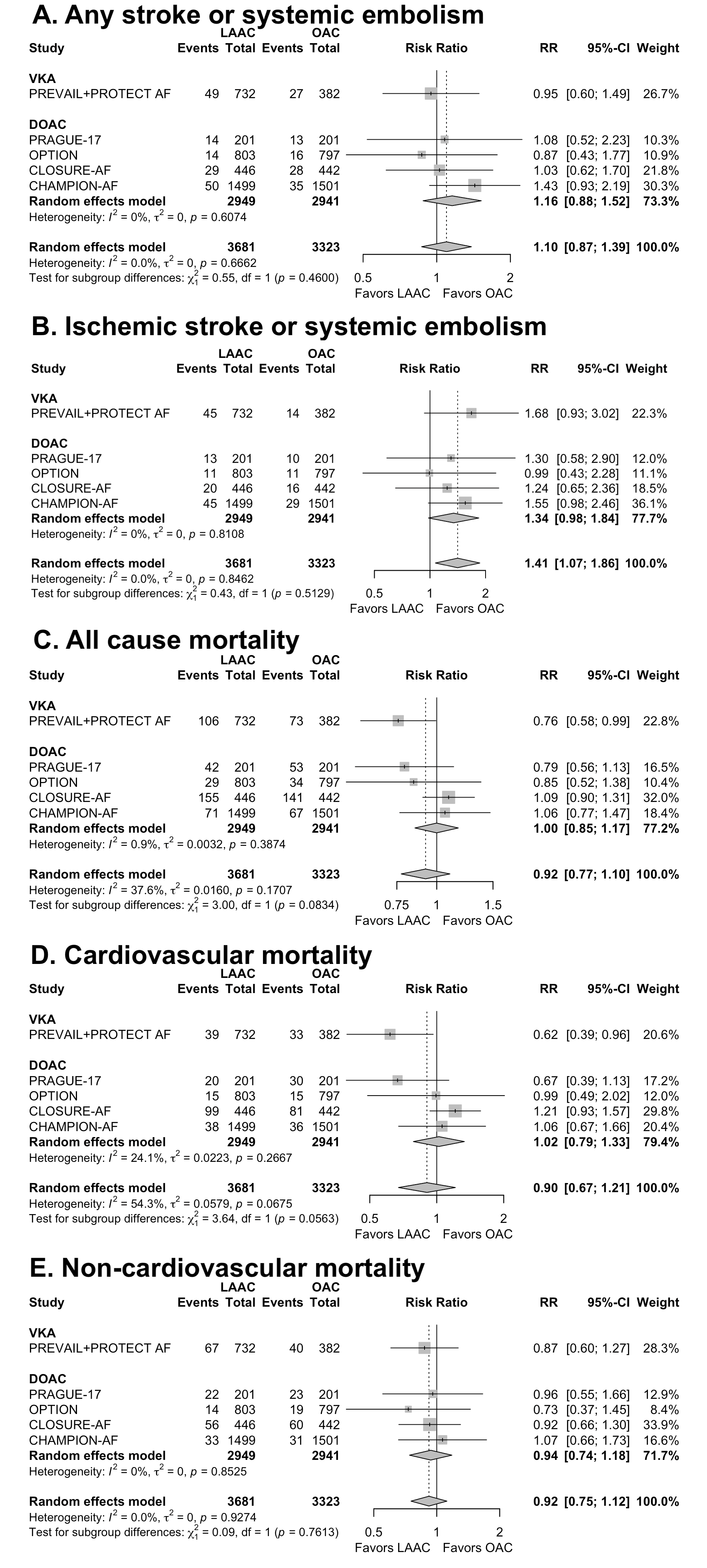


**Supplemental Figure 3**. Subgroup analysis of efficacy outcomes according to oral anticoagulant type. Forest plots show the effects of left atrial appendage closure versus oral anticoagulation on (A) any stroke or systemic embolism, (B) ischemic stroke or systemic embolism, (C) all-cause mortality, (D) cardiovascular mortality, and (E) non-cardiovascular mortality, stratified by comparator type (vitamin K antagonist (VKA) or direct oral anticoagulant (DOAC)). Squares represent trial-specific risk ratios, with area proportional to study weight, and horizontal lines indicate 95% confidence intervals; diamonds represent pooled estimates for each subgroup and overall. Values less than 1.0 favor left atrial appendage closure. P values for subgroup differences were derived from interaction testing. LAAC=left atrial appendage closure; OAC=oral anticoagulation; RR=risk ratio.


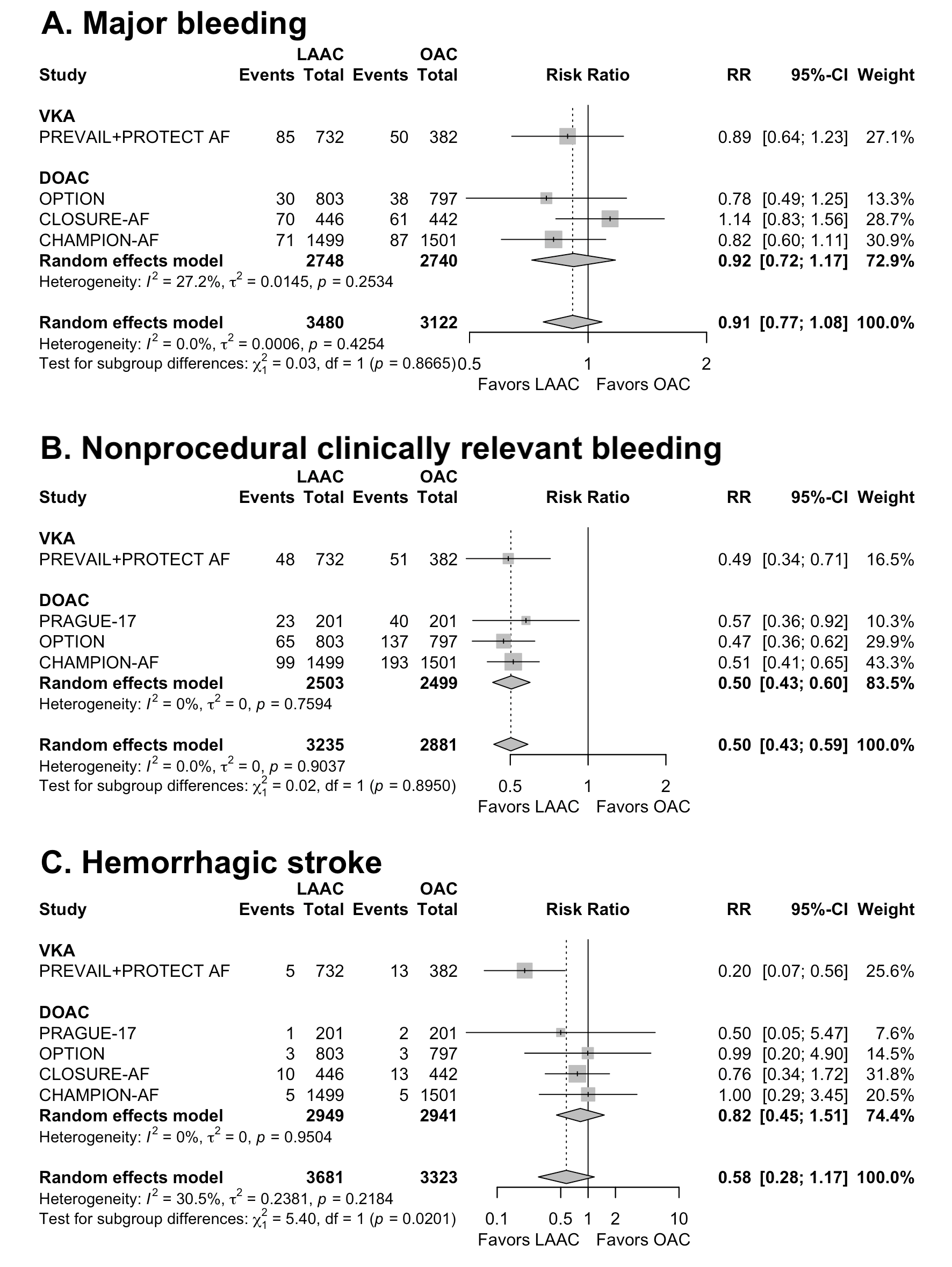


**Supplemental Figure 4**. Subgroup analysis of safety outcomes according to oral anticoagulant type. Forest plots show the effects of left atrial appendage closure versus oral anticoagulation on (A) major bleeding, (B) nonprocedural clinically relevant bleeding, and (C) hemorrhagic stroke, stratified by comparator type (vitamin K antagonist (VKA) or direct oral anticoagulant (DOAC)). Squares represent trial-specific risk ratios, with area proportional to study weight, and horizontal lines indicate 95% confidence intervals; diamonds represent pooled estimates for each subgroup and overall. Values less than 1.0 favor left atrial appendage closure. P values for subgroup differences were obtained from tests of interaction. LAAC=left atrial appendage closure; OAC=oral anticoagulation; RR=risk ratio; VKA=vitamin K antagonist; DOAC=direct oral anticoagulant.


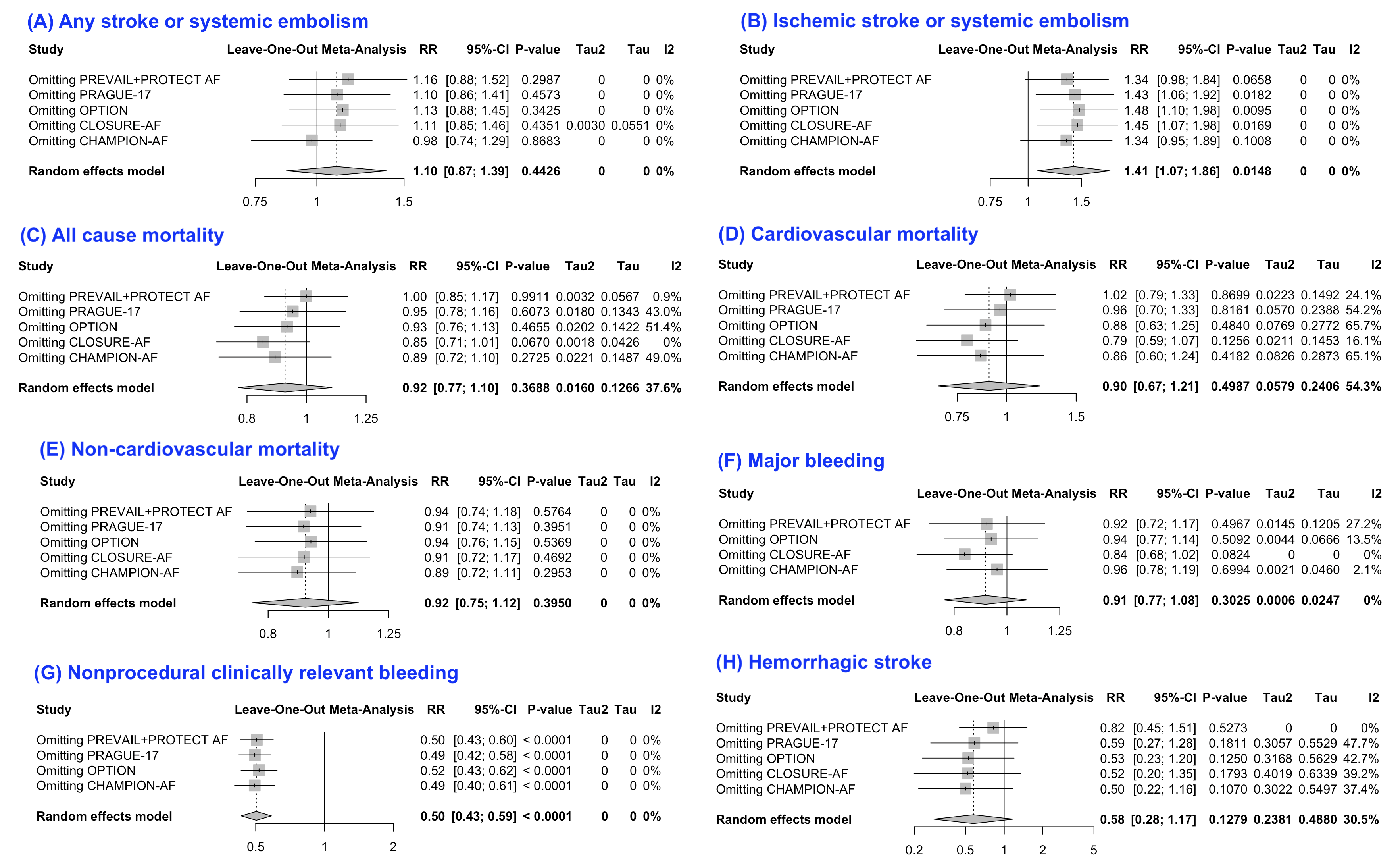


**Supplemental Figure 5**. Leave-one-out sensitivity analyses for efficacy and safety outcomes. Leave-one-out random-effects meta-analyses evaluating the influence of individual randomized comparisons on the pooled estimates for (A) any stroke or systemic embolism, (B) ischemic stroke or systemic embolism, (C) all-cause mortality, (D) cardiovascular mortality, (E) non-cardiovascular mortality, (F) major bleeding, (G) nonprocedural clinically relevant bleeding, and (H) hemorrhagic stroke. CI=confidence interval; RR=risk ratio.


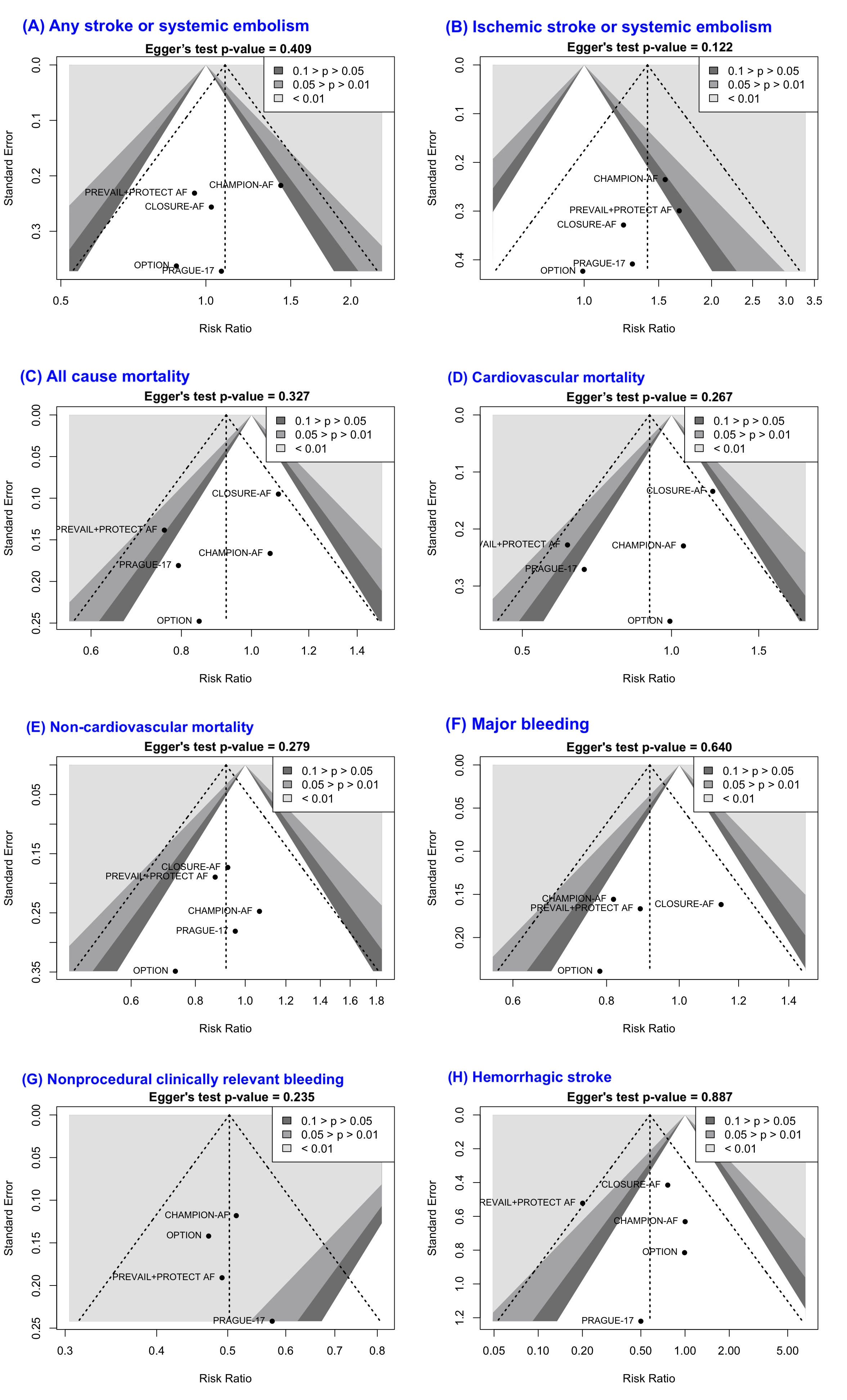


**Supplemental Figure 6**. Contour-enhanced funnel plots for assessment of small-study effects. Contour-enhanced funnel plots evaluating potential small-study effects for (A) any stroke or systemic embolism, (B) ischemic stroke or systemic embolism, (C) all-cause mortality, (D) cardiovascular mortality, (E) non-cardiovascular mortality, (F) major bleeding, (G) nonprocedural clinically relevant bleeding, and (H) hemorrhagic stroke.

# **Supplemental Table 6**. LAAC Procedural- or Device-Related Complications in Included Trials.

| **Outcome** | **PROTECT AF** | **PREVAIL** | **PRAGUE-17** | **OPTION** | **CHAMPION-AF** | **CLOSURE-AF** |
| --- | --- | --- | --- | --- | --- | --- |
| **Procedural- or device-related adverse events/total, n/N (%)^a^** | 37/463 (8.0) | 6/269 (2.2) | 4/201 (3.0) | 26/783 (3.3) | 53/1408 (3.8) | 24/421 (5.7) |
| **Pericardial effusion** | 22 | 1 | 0 | 3 | 9 | 5* |
| **Device embolization** | 3 | 2 | 1 | 0 | 0 | 1 |
| **Device-related thrombosis at follow-up imaging** | 6 | NR | 6 | 8 | 63/1320† | 14/320 |
| **Residual leak >0 to ≤3 mm at follow-up imaging, n/N (%)** | NR | NR | NR | 124/734 (17.6) | 205/1012 (20.3)† | 37/320 (11.6)‡ |
| **Residual leak >3 to ≤5 mm at follow-up imaging, n/N (%)** | NR | NR | NR | 8/734 (1.1) | 14/1012 (1.4)† | 19/320 (5.9)‡ |
| **Residual leak >5 mm at follow-up imaging, n/N (%)** | NR | NR | NR | 2/734 (0.3) | 0/1012 (0.0)† | 2/320 (0.6)‡ |

Abbreviations: NR, not reported.

a In the PROTECT AF, PREVAIL, and PRAGUE-17 trials, procedural- or device-related adverse events were categorized within 7 days post-procedure, and in the OPTION trial, within 10 days. In CHAMPION-AF, adjudicated periprocedural events were reported within 7 days after the procedure, whereas the overall device- or procedure-related serious adverse event total used in this table was site-reported through 3 years. In CLOSURE-AF, periprocedural complications were reported by day 7 after implantation or by hospital discharge.

† Four-month imaging time point in CHAMPION-AF.

* Pericardial tamponade in CLOSURE-AF.

‡ CLOSURE-AF leak categories were reported as <3 mm, 3-5 mm, and >5 mm at 3 months imaging follow-up.

# **Supplemental Table 7**. PRISMA 2020 Checklist

| **Section and Topic** | **Item #** | **Checklist item** | **Location where item**  **is reported** |
| --- | --- | --- | --- |
| **TITLE** | | |  |
| Title | 1 | Identify the report as a systematic review. | Title page (p. 1) |
| **ABSTRACT** | | |  |
| Abstract | 2 | See the PRISMA 2020 for Abstracts checklist. | Abstract (p. 1) |
| **INTRODUCTION** | | |  |
| Rationale | 3 | Describe the rationale for the review in the context of existing knowledge. | Introduction (pp. 3–4) |
| Objectives | 4 | Provide an explicit statement of the objective(s) or question(s) the review addresses. | End of Introduction (p. 4) |
| **METHODS** | | |  |
| Eligibility criteria | 5 | Specify the inclusion and exclusion criteria for the review and how studies were grouped for the syntheses. | Methods, Section 2.2 (pp. 4–5) |
| Information sources | 6 | Specify all databases, registers, websites, organisations, reference lists and other sources searched or consulted to identify studies. Specify the date when each source was last searched or consulted. | Methods, Section 2.1 (p. 4) |
| Search strategy | 7 | Present the full search strategies for all databases, registers and websites, including any filters and limits used. | Supplemental Tables 1–4 |
| Selection process | 8 | Specify the methods used to decide whether a study met the inclusion criteria of the review, including how many reviewers screened each record and each report retrieved, whether they worked independently, and if applicable, details of automation tools used in the process. | Methods, Section 2.4 (p. 5) |
| Data collection process | 9 | Specify the methods used to collect data from reports, including how many reviewers collected data from each report, whether they worked independently, any processes for obtaining or confirming data from study investigators, and if applicable, details of automation tools used in the process. | Methods, Section 2.5 (p. 5) |
| Data items | 10a | List and define all outcomes for which data were sought. Specify whether all results that were compatible with each outcome domain in each study were sought (e.g. for all measures, time points, analyses), and if not, the methods used to decide which results to collect. | Methods, Sections 2.3 and 2.5 (pp. 5–6) |
|  | 10b | List and define all other variables for which data were sought (e.g. participant and intervention characteristics, funding sources). Describe any assumptions made about any missing or unclear information. | Methods, Section 2.5 (p. 5) |
| Study risk of bias assessment | 11 | Specify the methods used to assess risk of bias in the included studies, including details of the tool(s) used, how many reviewers assessed each study and whether they worked independently, and if applicable, details of automation tools used in the process. | Methods, Section 2.6 (p. 6) |
| Effect measures | 12 | Specify for each outcome the effect measure(s) (e.g. risk ratio, mean difference) used in the synthesis or presentation of results. | Methods, Section 2.7 (p. 6) |
| Synthesis methods | 13a | Describe the processes used to decide which studies were eligible for each synthesis (e.g. tabulating the study intervention characteristics and comparing against the planned groups for each synthesis (item #5)). | Methods, Sections 2.2, 2.3, and 2.7 (pp. 4–6) |
|  | 13b | Describe any methods required to prepare the data for presentation or synthesis, such as handling of missing summary statistics, or data conversions. | Methods, Section 2.7 (p. 6) |
|  | 13c | Describe any methods used to tabulate or visually display results of individual studies and syntheses. | Methods, Sections 2.5 and 2.7 (pp. 5–6) |
|  | 13d | Describe any methods used to synthesize results and provide a rationale for the choice(s). If meta-analysis was performed, describe the model(s), method(s) to identify the presence and extent of statistical heterogeneity, and software package(s) used. | Methods, Section 2.7 (pp. 6–7) |
|  | 13e | Describe any methods used to explore possible causes of heterogeneity among study results (e.g. subgroup analysis, meta-regression). | Methods, Section 2.7 (p. 7) |
|  | 13f | Describe any sensitivity analyses conducted to assess robustness of the synthesized results. | NA (no sensitivity analyses prespecified or reported) |
| Reporting bias assessment | 14 | Describe any methods used to assess risk of bias due to missing results in a synthesis (arising from reporting biases). | Methods, Section 2.7 (p. 7) |
| Certainty assessment | 15 | Describe any methods used to assess certainty (or confidence) in the body of evidence for an outcome. | Methods, Section 2.8 (p. 7); Supplemental Table 6 |
| **Section and Topic** | **Item #** | **Checklist item** | **Location where item**  **is reported** |
| **RESULTS** | | |  |
| Study selection | 16a | Describe the results of the search and selection process, from the number of records identified in the search to the number of studies included in the review, ideally using a flow diagram. | Results, Section 3.1 (p. 7); Figure 1 |
|  | 16b | Cite studies that might appear to meet the inclusion criteria, but which were excluded, and explain why they were excluded. | Figure 1 |
| Study characteristics | 17 | Cite each included study and present its characteristics. | Results, Section 3.1 (pp. 7–8); Table 1 |
| Risk of bias in studies | 18 | Present assessments of risk of bias for each included study. | Results, Section 3.1 (p. 8); Supplemental Figure 2 |
| Results of individual studies | 19 | For all outcomes, present, for each study: (a) summary statistics for each group (where appropriate) and (b) an effect estimate and its precision (e.g. confidence/credible interval), ideally using structured tables or plots. | Figures 2–3; Supplemental Figures 3–4 |
| Results of syntheses | 20a | For each synthesis, briefly summarise the characteristics and risk of bias among contributing studies. | Results, Sections 3.1–3.5 (pp. 7–11) |
|  | 20b | Present results of all statistical syntheses conducted. If meta-analysis was done, present for each the summary estimate and its precision (e.g. confidence/credible interval) and measures of statistical heterogeneity. If comparing groups, describe the direction of the effect. | Results, Sections 3.2–3.4 (pp. 8–11); Figures 2–3 |
|  | 20c | Present results of all investigations of possible causes of heterogeneity among study results. | Results, Section 3.4 (p. 11); Supplemental Figures 3–4 |
|  | 20d | Present results of all sensitivity analyses conducted to assess the robustness of the synthesized results. | NA (no sensitivity analyses reported) |
| Reporting biases | 21 | Present assessments of risk of bias due to missing results (arising from reporting biases) for each synthesis assessed. | Not assessed because fewer than 10 studies were included; Methods, Section 2.7 (p. 7) |
| Certainty of evidence | 22 | Present assessments of certainty (or confidence) in the body of evidence for each outcome assessed. | Results, Section 3.5 (p. 11); Supplemental Table 6 |
| **DISCUSSION** | | |  |
| Discussion | 23a | Provide a general interpretation of the results in the context of other evidence. | Discussion, Section 4 (pp. 11–14) |
|  | 23b | Discuss any limitations of the evidence included in the review. | Section 4.1 (p. 14) |
|  | 23c | Discuss any limitations of the review processes used. | Section 4.1 (p. 14) |
|  | 23d | Discuss implications of the results for practice, policy, and future research. | Discussion, Section 4 (pp. 13–14); Conclusions (p. 15) |
| **OTHER INFORMATION** | | |  |
| Registration and protocol | 24a | Provide registration information for the review, including register name and registration number, or state that the review was not registered. | Methods, Section 2 (p. 4); Protocol statement (p. 15) |
|  | 24b | Indicate where the review protocol can be accessed, or state that a protocol was not prepared. | Methods, Section 2 (p. 4); Protocol statement (p. 15) |
|  | 24c | Describe and explain any amendments to information provided at registration or in the protocol. | Methods, Section 2: “adhered to without deviation” / “no departures from the original methodological plan” (p. 4) |
| Support | 25 | Describe sources of financial or non-financial support for the review, and the role of the funders or sponsors in the review. | No specific financial or non-financial support reported; Acknowledgements (p. 15) |
| Competing interests | 26 | Declare any competing interests of review authors. | Conflict of interest (p. 15) |
| Availability of data, code and other materials | 27 | Report which of the following are publicly available and where they can be found: template data collection forms; data extracted from included studies; data used for all analyses; analytic code; any other materials used in the review. | Data availability (p. 15) |

*From:* Page MJ, McKenzie JE, Bossuyt PM, Boutron I, Hoffmann TC, Mulrow CD, et al. The PRISMA 2020 statement: an updated guideline for reporting systematic reviews. BMJ 2021;372:n71. doi: 10.1136/bmj.n71

For more information, visit: <http://www.prisma-statement.org/>
